# At the coalface and the cutting edge: general practitioners’ accounts of the rewards of engaging with HIV medicine

**DOI:** 10.1186/1471-2296-14-39

**Published:** 2013-03-21

**Authors:** Christy E Newman, Asha Persson, John BF de Wit, Robert H Reynolds, Peter G Canavan, Susan C Kippax, Michael R Kidd

**Affiliations:** 1 National Centre in HIV Social Research, The University of New South Wales, Level 3 John Goodsell Building, Sydney, NSW, 2052, Australia; 2 Department of Modern History, Politics and International Relations, W6A, Macquarie University, Sydney, NSW, 2109, Australia; 3 Consumer advocate (independent) for people living with HIV in Australia, 14 Napalle Street, Warana, QLD, 4575, Australia; 4 Social Policy Research Centre, The University of New South Wales, Level 2 John Goodsell Building, Sydney, NSW, 2052, Australia; 5 Faculty of Health Sciences, Flinders University, GPO Box 2100, Adelaide, SA, 5001, Australia

**Keywords:** HIV, General practice, Workforce issues, Rewards, Australia

## Abstract

**Background:**

HIV has become a chronic manageable infection in the developed world, and early and lifelong treatment has the potential to significantly reduce transmission rates in the community. A skilled and motivated HIV medical workforce will be required to achieve these health management and prevention outcomes, but concerns have been noted in a number of settings about the challenges of recruiting a new generation of clinicians to HIV medicine.

**Methods:**

As part of a larger qualitative study of the HIV general practice workforce in Australia, in-depth interviews were conducted with 31 general practitioners accredited to prescribe HIV medications in community settings. A thematic analysis was conducted of the de-identified transcripts, and this paper describes and interprets accounts of the rewards of pursuing and sustaining an engagement with HIV medicine in general practice settings.

**Results:**

The rewards of initially becoming involved in providing care to people living with HIV were described as *interest and inspiration, community calling* and *right place, right time*. The rewards which then supported and sustained that engagement over time were described as *challenge and change, making a difference* and *enhanced professional identity*. Participants viewed the role of primary care doctor with special expertise in HIV as occupying an ideal interface between the ‘coalface’ and the ‘cutting edge’, and offering a unique opportunity for general practitioners to feel intimately connected to both community needs and scientific change.

**Conclusions:**

Approaches to recruiting and retaining the HIV medical workforce should build upon the intellectual and social rewards of this work, as well as the sense of professional belonging and connection which is imbued between both doctors and patients and across the global and national networks of HIV clinicians. Insights regarding the rewards of engaging with HIV medicine may also be useful in enhancing the prospect of general practice as a career, and strengthening retention and job satisfaction among the existing general practice workforce.

## Background

*HIV, I find, is a fascinating infection*. (GP_16)

The history of HIV medicine continues to feature remarkable turning points [1]. When the first cases were diagnosed in developed nations in the early 1980s, clinicians were called upon to contribute both specialist and generalist care in the acute management of HIV infection, particularly those relating to opportunistic infections, and all too often, palliative care [2]. The progressive introduction of combination antiretroviral therapy (ART) from the mid-1990s marked a significant milestone, although one accompanied by uncertainty about the best ways to make use of the new treatments [3,4]. HIV medicine today tends to be described – in developed nations at least – as chronic illness management, with a particular focus on ART delivery and monitoring alongside the management of a range of co-morbid and psychosocial impacts associated with living with HIV long term [5]. Recent research further suggests that timely treatment of HIV may have the potential to decrease rates of new infections in the coming years, which could again transform the role that HIV plays in shaping both public health agendas and the lives of affected communities [6,7].

In the early years of the HIV epidemic, considerable research attention was paid to the experiences of the clinicians who were critically involved in shaping and providing HIV medicine. For example, the impacts and stressors of providing acute and palliative HIV care were well recognised across the health and medical professions [8,9]. The workforce implications of the introduction of effective treatments in the 1990s also received attention, including speculation about what the transformation of HIV medicine might mean for the relationship between clinicians and people living with HIV (PLHIV) [10,11]. However, in more recent years, despite a complex new set of social, economic and political dynamics becoming apparent that will affect the engagement of the medical workforce with HIV medicine [12], only a limited amount of research has addressed HIV health workforce issues in the developed world [13-16]. For example, there is evidence to suggest that clinicians who were involved in providing HIV care from the ‘early years’ are now heading towards retirement [1,17-19] and that the recruitment and retention of trained clinicians may prove increasingly difficult [15,20-22], particularly in light of the more multidisciplinary and primary-care focused models of health service delivery being promoted in this new ‘chronic illness’ era of HIV medicine [1,23,24].

HIV medical care is accessed in a range of settings in Australia, including publically funded hospitals and sexual health centres, but a particular feature is the role of the skilled and accredited medical practitioner working in private general practice in the community [23,25-27]. [The medical specialty of general practice in Australia is similar to what is known as family medicine in some other countries, especially in North America.] The benefits of managing HIV in primary care settings have been recognised for some time in Australia, with general practitioners (GPs) permitted to pursue accreditation to prescribe HIV medications (restricted in Australia under Section 100 of the Highly Specialised Drugs Program) [2,28]. At the time of publication, an estimated 123 GPs were ‘active’ HIV s100 prescribers around Australia, with most participating in the accreditation programs operated by the Australasian Society for HIV Medicine (New South Wales, Victoria, South Australia, Australian Capital Territory), and the University of Queensland (Queensland) [29,30]. As approximately 25,000 GPs were providing health care in Australia in 2012 [31], this suggests that less than 0.5% of GPs were accredited to prescribe HIV treatments at that time, and the majority of those were based in central urban areas. These workforce distribution issues were noted in the Sixth National (Australian) HIV Strategy (2010–2013), with particular reference to the ‘fluctuating distribution of Section 100 prescribers … [and] the recruitment and retention difficulties for Section100 GP prescribers and clinicians with an interest in HIV’ [32]: 47]. More recent research confirms that as demand for HIV clinical services is increasing in Australia, supply is decreasing [33]. As ART becomes further consolidated as central to both the health of individuals living with HIV and the prevention of ongoing transmission in the community [7,34], the need for a greater number of skilled and motivated s100 GP prescribers is likely to continue to grow.

However, recent years have also seen an increase in the number of new infections in Australia, and a gradual increase in the proportion of people living with HIV who identify as heterosexual, and/or who were born overseas or have partners born overseas [35]. The geographic distribution of where PLHIV live shows a gradual dispersal to outer urban and regional areas, away from the traditional concentration in the main capital cities [36,37]. The effective treatment of HIV also means people are living longer, and are therefore facing a new range of health issues related to ageing with HIV [38,39]. In addition to these new challenges for the delivery of HIV clinical care, the Australian medical profession is itself undergoing transformation, particularly through the ageing, feminisation and internationalisation of the workforce [40]. This combination of factors has inspired a process of reflection and renewal amongst the professional and advocacy organisations working in this field regarding their understandings of and approaches to engaging clinicians with HIV medicine.

While there is almost no published research related this topic, the final report of a project conducted by the Australasian Society for HIV Medicine on ‘Models of Access and Clinical Service Delivery for HIV Positive People in Australia’ described key barriers to attracting and retaining graduate doctors to HIV or sexual health medicine as ‘limited postgraduate training opportunities, fear of the complexity of care required for those living with HIV (co-morbidities, drug toxicities and side effects), irregular support and mentoring of practitioners and incongruent financial benefits to the level of care required’ [23]: 72]. It should be noted that accreditation to become an s100 HIV community prescriber typically involves completing a training course (or having prior learning recognised), demonstrating knowledge of relevant state/territory guidelines, having access to appropriate support from senior clinicians working in a designated inpatient HIV unit in a public hospital, and demonstrating a commitment to ongoing HIV specific medical education [41]. This last criteria is often claimed to be a potential disincentive to both pursuing and maintaining HIV prescriber rights, as these continuing medical education requirements tend to be additional to the requirements for maintaining general practice accreditation [12]. Other barriers noted in this report to the retention of the existing cohort of high HIV-caseload s100 prescriber GPs include ‘ageing, work-life balance, inadequate remuneration and recognition and professional burnout’ [23]: 33].

Although the available literature on the involvement of family doctors in the care of PLHIV is typically too specific to the particular health system conditions and contexts in which they operate to be easily translatable to the Australian setting [42,43], there are general insights that are also worth noting here. HIV medicine has historically been the purview of specialist disciplines across much of the world, with the role of primary care in the prevention, diagnosis and management of HIV infection often unclear or contested [43]. The issue of ART prescribing appears to be one that is most differently managed in different developed world countries. In many countries, the initiation and monitoring of ART is restricted to specialist clinicians, with generalist care and coordination a shared responsibility with primary care clinicians [44,45]. UK GPs have been shown to support this distinction, having demonstrated little interest in pursuing what is perceived to be a complex and rapidly changing field of knowledge [16]. However, as the number of people on ART increases and their care needs become more chronic rather than acute, the potential value of better integrating primary care providers into the care of PLHIV has become increasingly recognised [1]. Research on the most effective models for sharing care between primary and specialist clinicians is limited, but evidence is now emerging on how quality of care can be improved by integrating primary care [1,46,47]. Much of this is literature is organised around the principle of establishing ongoing and trusting relationships between PLHIV and their primary care providers [48-51]. As Wong et al. recently put it, ‘GPs have advantages in providing HIV services because of their position as trusted, community-based, long-term advocates for their patients’ [52].

Inspired by the complexity and urgency of these health service issues, the research on which this paper reports sought to better understand the professional rewards for GPs of engaging with HIV medicine. A paper that we published from the first phase of data collection addressed some aspects of this, describing what a group of Australian policy key informants believed was ‘moving’ GPs to provide specialist HIV care in the community [12], with identified themes organised around the clinical, professional and political dimensions of the role of the HIV general practice doctor. More specifically, the willingness of individual clinicians to become involved was seen to be shaped by the representation or ‘social construction’ of HIV medicine; the balance between the professional opportunities and obligations offered by HIV medicine; and the ‘fit’ between the politics of HIV medicine and clinicians’ personal beliefs. The ASHM report mentioned above also speculated on professional motivations, surmising that ‘the many health professionals working in these areas of HIV medicine and with marginalised communities … remain there because of a personal commitment and passion. There is evidence that this situation will not continue indefinitely’ [23]: 25]. Our research sought to provide first-person accounts from the GPs who provide HIV care in Australia regarding what they believe motivates and sustains their engagement. What can be learnt from the perspectives of these experienced clinicians about the rewards of providing HIV care, and how might those insights be used to inform the engagement of a new generation of GPs and other primary health care practitioners with HIV medicine?

## Methods

The HIV General Practice Workforce Project was a three year study funded to explore the factors that shape GPs’ decisions to pursue the training and accreditation required to prescribe HIV medications in Australia, and to also build knowledge on the broader role of GPs in maintaining and enhancing the health of PLHIV. Data for the study included interviews with key informants [12,28] and with clinicians with experience in providing HIV care in different caseload and geographical general practice settings across Australia. Ethics approvals were received from the National Research and Evaluation Ethics Committee of the Royal Australian College of General Practitioners and the Human Research Ethics Committees of the participating universities.

This paper reports on the analysis of in-depth interviews conducted with 31 GPs who were (at the time of interview) actively engaged in providing HIV medical care in private general practice clinics and who had also received special accreditation to prescribe HIV medications in those settings. Participants self-selected (contacting the study coordinator to arrange an interview time) after they received information on the study via the electronic newsletters of the professional organisations representing Australian health and medical practitioners caring for people living with HIV, or via email distribution of the information flyer by members of the study’s Expert Advisory Committee (see Acknowledgements). The study team aimed to interview GPs based in different regions of Australia, both urban and rural locations, and in areas of high and low HIV caseload. We also aimed to achieve demographic diversity in the gender, age, and cultural profile of participants. However, to a certain extent, we were limited in our capacity to influence the study sample, because we were reliant on participants self-selecting rather than being directly invited to take part. Participants were advised that the semi-structured question guide was open-ended, and the interview questions would explore a range of issues relating to their personal and professional experiences, with particular reference to the field of HIV medicine. Before the start of each interview, written consent was secured from the participants who were interviewed face-to-face, and verbal consent (following a structured protocol) from the participants interviewed by phone. Interviews lasted between thirty minutes and two hours, with the average length of interview being around forty five minutes. All participants were offered AUD$150 reimbursement, in recognition of lost income for these GPs who were working in private practice settings.

The data analysis was informed by best practice guidelines for thematic analysis [53], which involved identifying recurrent patterns in the data and testing these through a process of constant comparison with variations within potential themes and then across the whole set of data. A coding framework was developed and discussed by the writing team, drawing on the areas of expertise of each researcher including HIV prevention, care and treatment, HIV patient advocacy and community histories, and experiences of living with HIV. One of the most consistent codes was that of ‘rewards’, and this captured data describing the motivations, satisfactions and passions attached to an engagement with HIV medicine. A second round of analysis then examined this data in more detail, resulting in six themes, each of which is discussed in detail in this paper. Rigour was established through an iterative process of discussion and revision, both within the research team and in consultation with members of the study’s broader Expert Committee, which also includes representatives of Australia’s peak HIV and general practice organisations. All interview extracts have been reproduced here with a numbered code to protect participant confidentiality.

## Results

### Participant profile

Interviews were conducted either in person (21) or by phone (10) between September 2010 and October 2011. Participants included 19 men and 12 women. Although not requested, more than half (n = 18) offered a description of their sexual identity as gay (n = 12) or heterosexual (n = 6). More than half the participants were aged 50 and over with an age range of 32–62 years; most (n = 24) self-identified as Caucasian and nine as Asian, European or Middle Eastern in cultural heritage. All but one received their medical training in Australia, with the one exception also trained in an English-speaking developed country. As a group, these participants were able to describe experiences of providing HIV care across each of the Australian states and territories, but mainly in New South Wales, Victoria, Queensland and South Australia. Almost all (n = 27) were based in urban metropolitan settings, with the remaining participants based in regional Australia. Just over half (n = 18) reported a high caseload of HIV positive patients (i.e., representing a significant component of their clinical work), with the remaining (n = 13) participants reporting a low or medium caseload. Participants first became involved in different decades of the thirty year history of HIV medicine: 1982–1989, following the start of the epidemic (n = 6); 1990–1999, the decade when effective combination HIV treatments were introduced (n = 12); and 2000–2008, when HIV had transformed into a serious, chronic but manageable condition in many developed nations for many people (n = 13).

### The rewards of pursuing an engagement with HIV medicine

#### Interest and inspiration

Scientific curiosity was a consistent theme in descriptions of the factors that motivated an initial pursuit of HIV medicine:

I became very good at death, palliative medicine … So that was pretty horrible. But that was, again, solved by the, [by] how interesting it just all was. You know, these most obscure infections that the infectious disease people wouldn’t see in their lifetime we’d be seeing day in and day out. (GP_02, started in the 1980s)

I was attracted to it because I love the science, primarily, of infectious disease and I love the science of virology and HIV. (GP_07, started in the 2000s)

It’s nice to be at the coalface of something and at the cutting edge as well. So you’re not only aware of the latest breaking drugs but you’re able to prescribe them to people. (GP_28)

HIV medicine is invested here with a sense of excitement and dynamism. This is ‘cutting edge’ science, with a constantly changing knowledge base. HIV is seen to represent an inspiring model of medicine, particularly for GPs with the opportunity of putting scientific knowledge into practice at the ‘coalface’. Even the horror and stress of the early years, when little was available in the way of treatment, is seen to be countered by ‘how interesting it just all was’.

Feeling inspired by others was also described as central to how this group of GPs became involved, particularly for those starting out in practices alongside more experienced clinicians:

When I started out as a GP registrar … my very first supervisor was an HIV prescriber. So that was sort of, it was like, “Oh wow, HIV!” So… very exciting, a bit daunting. (GP_08, started in the 2000s)

Having an enthusiastic and passionate mentor, as well as the other doctors, was important, to have that lead in time with people who knew a lot and were very supportive. (GP_12, started in the 2000s)

I think probably it was the enthusiasm of the other doctors … they just seemed like a really passionate group of people, about what they did. And that’s kind of inspiring. (GP_23, started in the 2000s)

The key words here are enthusiasm, passion and excitement. Clearly, having access to the support of clinicians who had found a way to maintain their interest and inspiration in HIV medicine over time was of critical importance in translating that initial flare of interest into a sustained series of decisions to pursue the field.

#### Community calling

A second theme was feeling ‘called’ to pursue HIV medicine by the communities most affected by HIV, which in Australia were (and are) mainly gay men. Among the 31 active-prescriber GPs we interviewed, 12 offered an unprompted description of themselves as gay (all of them men), and described a deep identification with the gay community:

My peers were dying, I guess, was the major influence there. And identifying with the community that was affected seems something that, you know, there wasn’t really much choice in *not* doing it. (GP_01, started in the 1990s)

I suppose also with my sexual orientation, there was always some risk that [but] for the grace of God, that could be me, the person there with HIV. So … that was another driver as well, that I could identify with these guys … outside of medicine … [I]f I wasn’t doing it, I would feel that I was letting the team down. (GP_16, started in the 1990s)

There is a consistent message being conveyed here, regarding how personal connection and loyalty to the gay community inspired a sense of responsibility to acquire clinical skills in HIV care and treatment, particularly in the early years of the epidemic.

Another aspect of this notion of feeling ‘called’ was described by other GPs who felt a sense of obligation to provide HIV care for people affected by social marginalisation and ostracism:

[People living with HIV have] had more than enough to deal with through whatever they’ve been through and they needed a safe place. They needed an understanding environment. They needed somewhere that they could feel comfortable. (GP_03, started in the 1980s)

I guess I felt quite strongly that there, you know, that gay people were marginalised enough as it was and here was a disease that sort of marginalised them further. (GP_24, started in the 1990s)

I’m not rightwing, [but] I do believe the Bible and I believe in the dignity of people and that people should be treated with respect. And I thought, “Well look, if I am a Christian who believes that people have a right to be treated with respect, then the least I can do is offer a GP service for people who may find it difficult to get a GP.” (GP_26, started in the 2000s)

These quotes suggest that it was not only gay doctors who felt moved by a sense of personal duty in responding to the ‘call’ for clinicians to help. While the notion of community obligation may have a different resonance for GPs caring for lovers, partners and friends, these extracts point to a broader range of motivations among clinicians who pursued specialist training in HIV medicine than only personal identification.

Several of the clinicians did note, however, that the politics of engagement in which doctors who are gay once felt obliged to get involved no longer apply, opening up new questions about the dynamics between sexual identity and choice of specialisation in general practice medicine. As one GP put it:

I’m not a gay male so I don’t have the, you know, the burning passion … But then I know a lot of gay men that don’t, so yeah … who’s going to look after HIV? (GP_29)

#### Right place, right time

Several GPs described their experience of becoming involved in HIV medicine as an unplanned confluence of events:

When HIV first came around, first of all some of my patients were affected. And also in the early stages, the hospitals didn’t want to have anything to do with HIV because there was a lot of stigma about HIV and AIDS. And so there were, well number one is there were too many patients for them to manage anyway. They couldn’t cope. And so we got to manage more and more of their patients’ care. (GP_10, started in the 1980s)

It was back in the mid eighties. I was just working in a country town and there was nobody else who did it and a friend of mine had HIV, and just one thing led to another, to another, to another, to another and there you go. (GP_27, started in the 1990s)

These GPs see the role of the doctor as being responsive to the needs of the patient in their community. Whether in urban or regional Australia, the GP is viewed as holding a professional obligation to recognise that a new health issue is at their doorstep, and that there is a gap in the available primary care medical services to address that new need.

Even though all of these GPs at some point made deliberate choices to become and stay involved in this field, particularly in pursuing training and accreditation to prescribe HIV medications, several drew upon the metaphor of an ‘historical accident’ to explain the initiation of this sequence of events:

I didn’t stop to think about it. It was like this, you know, tidal wave of stuff happened and we were there, and so we just did it … It’s all just been an accident. Because people often say to me, you know, “You’ve planned your career so well and blah, blah, blah,” and it hasn’t been planned. Nothing has been planned. (GP_11, started in the 1980s)

Really by sheer accident … I got in touch with a drug rep who … was just wondering if I was interested in doing any of the HIV medicine, because I think they were looking for doctors around this area … So I thought, “Okay. You know, I might look at it.” (GP_08, started in the 2000s)

It was completely by accident, of course … I’ve been in hepatitis C for many, many years and that was a natural progression when I realised I had some co-infected patients. And then this became very important when I started up with the current practice I’m working in, which has quite a few HIV patients. And the opportunity came my way to do the training. (GP_13, started in the 2000s)

This metaphor of ‘accident’ was expressed by clinicians who became involved in HIV medicine in the early years, with the introduction of effective treatments, as well as in the past decade. This suggests that for many clinicians, their experience of pursuing HIV medicine initially featured a considerable element of chance and serendipity, even if this field had latterly come to represent a significant and meaningful dimension of their working lives.

### The rewards of sustaining an engagement with HIV medicine

#### Challenge and change

The most common way of articulating the rewards of providing HIV care in general practice was to describe this as an ‘intellectually stimulating’ field of practice, and one which was consistently challenging:

The medicine’s quite demanding and requires a high degree of understanding. And I think that’s a very rewarding thing in itself to actually be able to master the difficult signs associated with the disease, per se, and the responses to the disease … I like having that intellectual challenge, you know, with HIV … Looking for the answer and working it out, and getting someone better. It’s good. (GP_06, started in the 2000s)

Look, medically it’s very rewarding. It is so interesting. It’s cutting edge … HIV news makes front page news and it’s a really interesting field to work in … So medically I find it very stimulating … because it does branch into every aspect of life … it’s a bit of sexual health, it’s a bit of public health, it’s a bit of medicine, it’s a bit of, you know, social work … [and] there’s a bit of fun involved. (GP_23, started in the 2000s)

These GPs valued the experience of feeling tested, of being required to think through the many and varied aspects of this condition in order to come up with ‘the answer’ for each of their individual patients. The medicine is constructed as fascinating, interesting, diverse and stimulating, and these characteristics are linked to the belief that it is ‘good’ and ‘rewarding’ and ‘fun’ to feel pushed to the limits of knowledge, to know that the intellectual demands of the work are a challenge.

The second aspect of this theme drew upon a metaphor of ‘constant change’ in relation to the field of HIV medicine. For GPs involved from the early years of the Australian HIV epidemic, this was described as the experience of literally living through the making of medical history:

I’ve been [involved] since the very beginning. And like no other major health advance which has resulted in peoples’ lives being saved, you help the generation that were sick. It isn’t like, you know, curing polio or curing other infectious diseases in the past where you stopped the next lot of patients getting it; you actually turn the lives around of the people who have the infection. (GP_02, started in the 1980s)

I can’t think of one other disease that it’s been so rewarding to be involved with, in a way, because I mean we’ve had a turnaround that you would never have envisaged. And I mean that’s been, that’s been the exciting thing about it. (GP_30, started in the 1980s)

All of these clinicians have had the opportunity to work at the ‘coalface’ of the response to HIV, but describe this as having far more significance than only managing individual patients. These narratives place the community HIV prescriber at the forefront of history, contributing to a major new turn in scientific knowledge, and having firsthand experience of giving new life to a generation of patients.

GPs who became involved since the introduction of effective treatments in the mid to late nineties were more likely to describe feeling drawn to the rapid pace of scientific change in this field:

It’s such an interesting area to work in and such a rapid turnover … things are always changing … You know, they’ll often say by the time an HIV textbook comes out, it’s already a little bit out of date. (GP_08, started in the 2000s)

It is challenging because it’s such a fast moving area. You know, there’s plenty of challenge there. And that I think is an attraction if you want challenge. (GP_26, started in the 2000s)

HIV medicine’s intellectually stimulating. It’s a new field. There’s always new stuff coming through. It keeps you a bit on your toes. (GP_24, started in the 1990s)

This group of GPs, some of whom have been engaged with HIV medicine for up to thirty years, described the breadth of knowledge and pace of change in this field as providing a rewarding experience for the GPs who practice it. This was often based upon feeling intellectually stretched – being ‘kept on your toes’ – but there is also a persistent sense here that these community-based GPs feel intimately connected with the unfolding of new knowledge over time, of being part of history.

#### Making a difference

Another reward of providing HIV care in general practice was described as making a genuinely significant contribution to making and keeping patients well. This was sometimes contrasted with the days before effective treatments were available:

I was very fortunate to be around a lot of people we diagnosed way back in the early eighties who are still with us and it’s been my privilege, if I can say that, to be there for them but equally still looking after them now … how lucky can you be? (GP_03, started in the 1980s)

The medication [generated] a lot of hope and a lot of potential to say to someone, “Hey, I can change your life.” And that is a really fantastic feeling. And reflecting with patients when they were in [intensive care] and now they’re back at work and stable is, doesn’t happen that often in other areas of medicine. (GP_20, started in the 1990s)

A key dimension of contributing to successful patient outcomes was identified as the continuity of care that is made possible by being based in community-based health service settings:

It’s lovely because you sort of grow and age with them and get to know them quite well. Seeing the medications get better and seeing people live much longer than you thought were going to is very rewarding. (GP_01, started in the 1990s)

And because there’s that continuity of care, you have the luxury of seeing people on a regular basis, an ongoing basis … [and] you can sort of explore a bit more of their past history, you know, family history … work history, social history … that specialists might not actually get into … You really get to know them. (GP_08, started in the 2000s)

Travelling the road with patients… it’s been rewarding in seeing people change and evolve, and accept their HIV, and partnerships. And in women have children and become pregnant, and have uninfected children and relationships. (GP_31, started in the 1990s)

These quotes suggest that there is mutual benefit to be gained through building close and longstanding relationships between doctor and patient in this setting. The GPs get to know and ‘travel with’ their patients in a more sustained way than is perhaps typical, witnessing change and evolution in people’s lives over time, while patients have the opportunity to receive care from someone who can appreciate the fuller and more complex picture of their concerns and priorities.

Providing the main point of connection and coordination was a key dimension of the rewards these GP described, with an understanding that this again required an appreciation of complexity:

Being involved [with] patients with a chronic condition, managing them holistically … has been a really rewarding sort of experience … that model of chronic care, being the care coordinator of the patients … I think is rewarding. (GP_10, started in the 1980s)

I’m often the person that will be delivering a diagnosis. And for me it’s a privilege to be able to give that diagnosis and then say, “But … I can help you to look after this and we don’t have to send you off to someone else” … So it can all just be … like a one stop shop for me, for my patients. (GP_28, started in the 2000s)

Many of our participants valued this experience of being a ‘one-stop shop’ or ‘drawing the dots together’, and viewed this as essential to making a difference in patients’ lives in the context of what could sometimes be a quite complex set of health and social issues.

#### Enhanced professional identity

The final reward articulated by these GPs related to professional identity, with a particular focus on the potential for increasing job satisfaction through pursuing a special interest in HIV medicine:

Being stuck in general practice with, it’s either coughs and colds or a lot of chronic illness. And if you feel a bit specialised … particularly if other people perceive it as difficult, I think there’s some kudos in that. (GP_11, started in the 1980s)

I think you sort of feel a little bit like a sort of a ‘mini-specialist’ if you like. You feel like you, it’s a sub specialty and it sets you apart a little bit from the other GPs. And being able to be an s100 prescriber gives you more sort of power and rights, and so on. You feel like, “Okay, I’m a little bit different.” (GP_08, started in the 2000s)

I’m so glad I’ve got this as a focus … I think I’d be really tiring of general practice now if it wasn’t for this focus. So I’m really grateful to have found it. (GP_04, started in the 2000s)

For these GPs, HIV medicine offered a way to ‘moderate’ the more quotidian dimensions of general practice by developing a set of particular skills and areas of clinical expertise. This is seen to generate ‘kudos’, to set the HIV GP apart from others, to feel ‘a little bit different’. Importantly, these processes are also seen to be strengthening their capacity to remain engaged with general practice over the course of their medical careers.

Also strengthening the professional identity of the GP was the satisfaction they gained from feeling they were providing a service to a group of patients who may be marginalised and underserved:

I get a level of satisfaction from providing a service that people can’t access any other way … So I suppose right back to my radical student days I’m looking after the people that fall off the edges of the other services. (GP_21, started in the 1980s)

I look at my peers and it’ll come back to money, [but] generally doctors working in this area, my experience is we’re on a lower salary and much is done through genuine care for our patients. (GP_20, started in the 1990s)

This field I think really brings in really disadvantaged patients … And I don’t know if other fields of medicine can do what this field of medicine does in terms of helping people. (GP_05, started in the 2000s)

These extracts construct the role of the GP as one which is perfectly placed to facilitate both medical and social change. It is interesting to note that GPs who became involved in this area of practice at different points of the epidemic shared this investment in the social justice dimensions of the role.

Finally, there was a consistent theme expressed throughout these interviews on the collegiality and support which could be accessed through professional networks within the HIV sector:

[T]he collegiality of the area is far and away what’s kept me in it … I think it’s all a bit of a club and we’re all on the same side. And we share knowledge and we debrief. (GP_31, started in the 1990s)

I wouldn’t know a handful of general practitioners from my area but I know about fifty HIV GPs because we all see each other a lot. And … that’s a very nice part I think. There’s a bit of a club feel. (GP_30, started in the 1980s)

[I]t’s also rewarding to be involved with other health professionals that are highly motivated to engage in an area of challenge and significant difficulty… I think there’s quite a degree of collegiality or camaraderie amongst fellow prescribers. (GP_06, started in the 2000s)

There is clearly a deep and enduring sense of professional connection that has developed over time in this field, at least among the GPs we interviewed, who felt they formed a part of the ‘sub-speciality’ of HIV prescribing in general practice. Some of the features of this ‘club’ include collegiality, motivation and interest, a willingness to share information and support each other, and an ease in developing trust and camaraderie. This was described as a typical in general practice, and as something to particularly cherish and appreciate.

## Discussion

This qualitative analysis described a series of professional rewards which can be seen to shape the capacity and willingness of general practice doctors to provide care to people living with HIV today, in an era of chronic disease management. The themes which were identified in these interviews provide an account of the rewards that both inspired and sustained these GPs in engaging with HIV medicine as an area of special interest, and enriched their experience of general practice more broadly. Our use of qualitative methods means our findings cannot be claimed as generalisable, and our focus on the Australian setting somewhat limits its relevance to other developed nations, particularly those where HIV medications are relatively accessible and affordable. However, despite the particular focus of our study data, we believe there are important insights to be gained here regarding the rewards of engaging with HIV medicine in a broader range of health service and country settings, as well as for enhancing the value of the medical specialty of general practice as a career.

Our analysis suggests that, for these GPs, the rewards of initially pursing HIV medicine were ‘interest and inspiration’, ‘community calling’ and ‘right place, right time’. These findings partly reflect what Gerbert et al. have described as ‘the dual faces of passion: challenge and calling’ among physicians with HIV expertise in the United States [54]. In their paper, HIV clinicians are described as being motivated by a passion for both the science of HIV and for serving the populations most affected, and this does indeed seem to complement what our participants were suggesting. The idea that clinicians might be motivated to pursue particular areas of professional interest because they are interested in the science or connected to the populations affected is not new. For example, research conducted before the introduction of combination ART reported that clinicians who had a personal connection to patients or friends who were gay and/or HIV-positive reported an increased willingness to care for PLHIV [55-57]. However, what is new in our analysis is the additional or alternative explanation that sometimes professional passion can be inspired through the serendipitous happening upon a new and unexpected area of medical need. This possibility suggests that focusing attention only on those clinicians who have a burning desire to pursue this work, for either intellectual (e.g., ‘I love the science of virology and HIV’: GP_07) or socio-political (e.g., ‘I’m looking after the people that fall off the edges’: GP_21) reasons, may limit the range of opportunities for new and continuing professional engagement in this field, particularly among medical students and practitioners who may feel less clear about where their passions might lie. As noted in recent research, the reasons why doctors find particular specialities attractive or not are incredibly diverse, which should be viewed as enriching our understanding of medical workforce engagement, rather than complicating it [58]. Aiming to achieve workforce diversity as not only a social ‘good’ but also to enhance understanding of professional motivations in general practice opens up rather than closes down new opportunities for engagement.

The rewards that sustained engagement with HIV medicine over time were described by our participants as ‘challenge and change’, ‘making a difference’ and ‘enhanced professional identity’. What becomes clear in this analysis is that little significance was explicitly placed – by these clinicians at least – on financial or other economic incentives for providing HIV care. While beyond the scope of this paper, a range of challenges were also identified by these participants as barriers to caring for PLHIV in general practice settings. In particular, reduced remuneration for longer consultations was described as something that most of these GPs had come to believe was an unavoidable cost of choosing to be an HIV prescriber. It is interesting to note, therefore, that an alternative, more social, rendering of the concept of rewards was articulated here, whereby the choice to pursue HIV medicine was viewed as providing the clinician with a deeper sense of professional purpose and meaning than financial reward alone could provide. We can assume that this would have been shaped at least in part by the desire to present a positive self-image in the research interview, particularly in the participants’ deliberate contrasting of their own philosophies of doctoring with what they perceive as a ‘dollar-driven’ approach. Nonetheless, it is useful to recognise that in the case of HIV medicine, the recruitment and retention of clinicians may be strengthened if a broader, more socially organised, conceptualisation of the personal and professional rewards of this work is promoted.

In the absence of a sustained body of research on engaging clinicians with HIV medicine, the literature of most relevance to our findings is that which seeks to understand what motivates medical students and trainees to pursue general practice medicine as a career. General practice tends to be one of the fields of medicine which has faced persistent workforce shortages, and this has certainly also been the case in Australia, particularly in rural and remote areas [59,60]. Many studies have been conducted around the world in recent years to understand the factors that might more successfully promote general practice as a satisfying and rewarding medical career [61-66]. Although there is some variability, general practice (and family) medicine is often reported in this literature to hold lower levels of interest and prestige in the eyes of medical students than the other speciality fields [67]. Particular barriers compound these more generalised concerns about the image of general practice; for example, a recent survey of Australian general practice trainees identified increasing bureaucracy, workforce shortages, and poor remuneration as potential deterrents to a future career in general practice [68]. A conclusion often reached in this literature is that the attractions and rewards of general practice need to be better articulated and promoted to those who might consider joining the profession [69,70].

To that end, it is useful to consider how the rewards of engaging with HIV medicine identified in this paper might inform more general understandings of the rewards of pursuing a career in general practice. The first such contribution relates to the ‘intellectual’ rewards of general practice. Not only did many of the GPs we interviewed characterise HIV medicine as intrinsically interesting, they also viewed the ‘challenge and change’ involved in keeping up with scientific and clinical developments in this field as one of its major rewards. These accounts provide a counter perspective to the research literature which has consistently reported that medical students view general practice as offering insufficient ‘scientific-technical interest’ [63] or ‘intellectual content’ [71]. For example, a qualitative synthesis of the literature on choosing general practice reported a recurrent belief that general practice was less intellectually challenging than other areas of medicine, tending to treat only ‘common disease’ [67]. The evidence that our participants achieved great professional satisfaction in acquiring particular and additional forms of expert knowledge on the management of HIV infection challenges the perception that general practitioners and other primary care physicians manage the least exciting aspects of medical work. On the contrary, the GPs accredited to prescribe HIV medications describe their work as both ‘at the coalface’ *and* ‘cutting edge’. Shadbolt and Bunker have argued that ‘although flexibility and work-life balance are important motivators [of choosing general practice], more important is the *perceived intellectual challenge of the career’*[66]: italics added]. With this in mind, we propose that GPs with a special interest in HIV medicine provide an exemplar case to demonstrate how general practice can typically feature both ‘interest and inspiration’ and ‘challenge and change’, and can offer clinicians the opportunity to develop particular areas of interest flexibly over time.

Our second contribution relates to the ‘social’ rewards of general practice. The GPs we interviewed were keen to stress how much they valued ‘making a difference’ in the lives of their HIV positive patients, particularly in providing continuous and ‘whole-person’ care – including the prescription of restricted pharmaceutical treatments – in the community. This way of conceptualising the role of general practice is by no means unique to the management of HIV. As has been reported in research on chronic illness care: ‘GPs saw themselves as coordinators of care as well as advocates for patients, including educating them about their illness, helping them to understand specialist recommendations and working in partnership with them’ [72]: 31]. Medical trainees who decide to pursue general practice have also been shown to prioritise ‘variety’ and ‘continuity of care’ as major motivating factors in their decision [73]. And continuity of care was also recognised in Canadian research on the rewards of general practice, in addition to ‘having relationships with patients and their families’ and ‘being an immersed witness to the human condition’ [70]. This brings us to our next contribution, which is to suggest that the rewards of HIV care work also demonstrate that engaging with a special interest can have social or political impacts in addition to clinical and public health outcomes. Clinicians drawn to general practice have been shown to typically feature a ‘patient orientation’ [62], or ‘social orientation’ [66], and to feel a deep pride in their ‘social mission’, and in ‘making a greater contribution to society’ [74]: 142]. Our research suggests that this ethos of social engagement can also develop into a strong and long-term alliance between a community affected by a particular illness and the group of clinicians who care for them.

The third contribution relates to the ‘professional’ rewards of general practice. These GPs valued the relationships they were able to develop through HIV medicine, not only with patients, but also with peers. The sense of professional belonging and connection imbued among the community of HIV medical practitioners was viewed as uniquely traversing geographical and demographic differences. However, these rewards were very much seen to be associated with pursuing a ‘special interest’ in HIV medicine, and gaining ‘special rights’ to prescribe HIV medication, attracting ‘kudos’ in a medical community that is still seen to ascribe a greater degree of prestige to specialists [75]. Although research from Australia has found moderate to high rates of job satisfaction among GPs [76-78], the international research has often reported a pattern of longterm decline [79,80]. Much has been made in the UK [81,82] and Australia [83,84] of the potential for the model of ‘General Practitioner with Special Interests’ (GPSI) to challenge this by increasing the intellectual rewards of general practice work for the existing workforce and enhancing the prospects of a satisfying career in general practice for interested students and trainees [81,85]. What has been less well documented is the potential for GPSIs to also create new opportunities for professional networking and collaboration, organised around a shared set of intellectual, social and professional priorities which extend beyond the parameters of general practice medicine. Having regular contact with likeminded colleagues has been shown to increase job satisfaction among GPs [86], particularly those based in urban areas who are more likely to view competition as a barrier to professional networking [87]. Supporting the career development of GPs has also been argued to be essential for retaining mid- to late-career clinicians [88]. And it has been repeatedly shown that interactions with senior doctors have a major influence on choice of specialty for medical students [61,89,90], including those who end up choosing general practice [67,91]. We also know that one the most consistent rewards for GPs in their experience of this field of medicine is in teaching and sharing knowledge and gaining experience in mentoring [70]. Therefore, we feel that our findings confirm that celebrating and supporting opportunities for this kind of mutual exchange between senior and junior doctors, and the creation of a ‘sub-speciality’ camaraderie between likeminded clinicians, is therefore likely to not only increase the numbers of GPs willing to pursue and sustain their engagement with HIV medicine, but also those committed to pursuing a career in general practice more broadly.

Our analysis also provides a useful set of insights for contemporary health policy. The transformation of HIV from an acute to a chronic illness in most developed nations, and the associated policy shift to favour a greater proportion of HIV care being provided in community rather than specialist or hospital settings, will require clinicians to be willing and able to take on the work of providing that care. Our analysis provides guidance for ways to promote this work and engage a new generation of clinicians with HIV medicine. In addition, HIV treatment is becoming increasingly viewed as essential to not only the long-term health of HIV-positive people, but also as a (proposed) component of preventing the ongoing transmission of HIV infection. The emerging evidence, in combination with recent guidelines in the United States, is currently propelling a move towards ‘treatment-as-prevention’, based on the notion that antiretroviral treatment (ART) substantially reduces the risk of HIV transmission and, particularly when introduced early, may significantly affect the course of the HIV epidemic in a community [6]. Much debate is ongoing internationally regarding the potential and challenges of treatment for HIV prevention [92-94], and it is yet to be seen what this might imply for the methods and sites of HIV care delivery in the future. Increasing attention will have to be paid in coming years to the ways that HIV treatment is made available to those who need it, and this includes the capacity and willingness of skilled primary health care professionals to facilitate and support the evolving role of ART, particularly in general practice settings. This paper contributes to these debates by proposing that a focus on the rewards of engaging with HIV medicine can strengthen the capacity of primary care systems to deliver HIV care and treatment, and to enhance more broadly the professional cultures and collaborations between HIV and general practice medicine.

## Conclusion

The interviews we conducted with GPs suggest that an engagement with HIV medicine enables clinicians to develop strong and long-term relationships with and expertise about the care needs of people living with HIV ‘at the coalface’, while also feeling connected with a broader network of medical practitioners and other professionals concerned with and contributing to the ever-changing world of science: ‘the cutting edge’. The general practice HIV prescriber is being modelled here as the interface between these two worlds, offering a rewarding opportunity for general practitioners to feel intimately connected to both community needs and scientific change.

## Authors’ contributions

CN led the conception and design of the study, conducted or coordinated all forms of data collection and analysis, and led the drafting and revision of the manuscript. AP contributed to the analysis of the project data, and provided critical input into the drafting and revision of the manuscript. JdW participated in the design of the study, advised on all stages of data collection and analysis, and contributed to the drafting and revision of the manuscript. RR advised on the design of the study, the collection and analysis of data, and contributed to the drafting and revision of the manuscript. PC advised on the design of the study from the particular perspective of a consumer advocate for people living with HIV/AIDS, advised on the collection and analysis of data, and contributed to the drafting and revision of the manuscript. SK advised on the design of the study, the collection and analysis of the data, and contributed to the drafting and revision of the manuscript. MK participated in the design of the study, advised on all stages of data collection and analysis, and contributed to the drafting and revision of the manuscript. All authors read and approved the final manuscript.

## Authors’ information

CN (BA, PhD) is Senior Research Fellow at the National Centre in HIV Social Research, in Sydney, where she conducts qualitative research on social aspects of health and health care, with a particular focus on the lived experience and clinical management of HIV. Her current research explores the needs and experiences of the health and medical workforce in general practice settings.

AP (BA, PhD) is a social anthropologist and Research Fellow at the National Centre in HIV Social Research at The University of New South Wales. Since 2001, her research has primarily focused on ‘living with HIV’, including living heterosexually with HIV, children growing up with HIV, couples with mixed HIV status, and cultural discourses of health and illness.

JdW (MSc, PhD) is the Director of the National Centre in HIV Social Research and is an international expert in the social psychology of health and sexuality, with a particular contribution to the field of HIV prevention. His cross-disciplinary research interests encompass basic and applied social science research and focus on strengthening theory-informed and evidence based understandings of health promotion policies and programs.

RR (BA, PhD, MCouns) is an Associate Professor of Modern History and Associate Dean of Higher Degree Research in the Faculty of Arts, Macquarie University, Sydney. He is the author of two monographs on Australian gay life post 1970 and a psychotherapist in private practice.

PC (Dip. Comm Services) is an independent consumer advocate for people living with HIV in Australia. He has been closely involved in HIV education and advocacy since the start of the Australian epidemic. He has held a variety of positions over many years with the peak-organisation National Association of People Living with HIV/AIDS, including as President.

SK (BA, PhD, FASSA) is a social researcher of international standing with over twenty years’ experience and an extensive track record in sexuality and illicit drug use research and, more generally, the interface between social aspects of health and illness and clinical practice. She is a Fellow of the Academy of the Social Sciences in Australia.

MK (AM MD FRACGP) is a General Practitioner, Executive Dean of the Faculty of Health Sciences (including the School of Medicine) at Flinders University in Adelaide, South Australia, and Chair of the Australian Government’s Advisory Committee on Blood Borne Viruses and Sexually Transmissible Infections.

## Pre-publication history

The pre-publication history for this paper can be accessed here:

http://www.biomedcentral.com/1471-2296/14/39/prepub
